# Survival from bladder cancer in England and Wales up to 2001

**DOI:** 10.1038/sj.bjc.6604600

**Published:** 2008-09-23

**Authors:** P Whelan

**Affiliations:** 1 Pyrah Department of Oncology, St James' Hospital, Beckett St, Leeds LS9 7TS, UK

## Definition and clinical presentation

In 1998, a WHO consensus conference defined urothelial cancers and the preferred synomyn, urothelial cell carcinoma (UCC), in place of transitional cell carcinoma (TCC) (Epstein *et al*, 1998). Urothelial cell carcinoma accounts for up to 90% of malignant bladder tumours in the United Kingdom. The remainder comprise squamous cell carcinoma, adenocarcinoma and neuroendocrine tumours. Mesenchymal tumours such as sarcoma and haematological-based ones such as lymphoma are rarely found in the bladder, whereas adenocarcinoma from other pelvic organs (prostate, ovary, endometrium and rectum) may on occasions develop a true metastatic secondary lesion rather than problems from direct extension.

Tumour grade is associated with tumour stage, disease behaviour and prognosis. It has been traditionally defined G1–G3 by several systems based upon cell anaplasia and while this WHO definition and those from 1979 and 1992 will have operated during this period, separate systems were accepted by North America and Scandinavia as well. As a result, comparison of outcomes by stage has been problematic. In 1998, the WHO consensus classification introduced the term ‘Papilloma of low malignant potential’, equivalent to G1 (in Ta TCC the old WHO system), and low- and high-grade papillary UCCs, equivalent to G2 and G3 TCC, respectively. It was proposed that these terms would more closely predict disease behaviour; however, their usage remains controversial and will not have impacted on the period under study. The TNM staging of bladder cancers is based on the depth of invasion of the bladder wall (T), involvement of regional lymph nodes (N) and the presence or absence of metastases (Sobin and Wittekind, 2002).

Visible (macroscopic or frank) haematuria is the most common presenting sign, occurring in approximately 75% of patients. It is usually intermittent and painless. On investigation of male patients at high risk (over 50 years) presenting with symptoms of painless haematuria, between 20 and 25% are found to have bladder cancer. A more difficult problem is presented with invisible (microscopic) haematuria, which is associated with bladder cancer in around 6% of men in cohort studies. Unfortunately, 20% of male patients in the at-risk population are found to have invisible haematuria from a variety of causes, and in the same age group, up to 40% of women will have invisible haematuria, demonstrating the large prevalence of invisible haematuria (Khadra *et al*, 2000). Currently, there are no reliable tumour markers for the detection of bladder cancer, and as a consequence, all patients found to have significant invisible haematuria are investigated to exclude cancer. Approximately 20% of patients do not present with either visible haematuria or invisible haematuria but have irritative voiding symptoms such as frequency and urgency. The persistence of these symptoms following exclusion of a cause such as urinary tract infection requires further assessment.

During the 20 years under study, significant advances have been made in understanding the molecular pathology of TCC and the differences in the way superficial and invasive tumours may arise (Mitra *et al*, 2005). A number of genetic changes have been identified but tumours with a low clinical risk for progression to muscle-invasive disease show very little evidence of genetic instability. In contrast, invasive tumours harbour many genetic alterations (Kim and Quan, 2005; Wu, 2005; Knowles, 2006). Mutations of the FGF receptor 3 in the absence of TP53 mutations are frequent in low-grade papillary tumours. Deletions of chromosome 9 are common in both invasive and non-invasive cancers, and TP53 is the most frequently inactivated tumour suppressor gene in invasive tumours. Recent evidence suggests that oncogene-driven cell division cycles trigger the activation of DNA-damage response in the early stages of tumorigenesis (Bartkova *et al*, 2005).

Although the molecular events that characterise bladder cancer are increasingly defined and our understanding of relevant pathways and networks has evolved, it remains surprising that no significant markers either for diagnosis of superficial or invasive disease, let alone a marker able to define recurrence, or relative prognosis have gained widespread acceptance and consistent validity.

## Pathology and its relationship with prognosis

The term superficial bladder cancer encompasses a spectrum of disease that includes true superficial papillary lesions, which are of relatively low clinical risk, as well as disease extending into the lamina propria, which is at high risk of progressing to muscle-invasive bladder cancer (MIBC). The majority of cancers at presentation (70%) are confined to the urothelium (Ta) or have invaded the sub-epithelial connective tissue of the lamina propria (T1). The paradox in which a spectrum of disease is termed ‘superficial’ yet encompasses cancers that have low and high risk for recurrence and progression and that have divergent molecular pathways has resulted in a change of terminology from ‘superficial bladder cancer’ to ‘non-muscle-invasive bladder cancer’ (NMIBC). The term NMIBC more correctly describes the spectrum with its diverse clinical and pathological phenotype.

A variant of NMIBC, carcinoma *in situ*, is most commonly seen in association with either high-grade Ta, T1 lesions (20%), and in MIBC. In approximately 5% of cases, carcinoma *in situ* occurs in isolation (primary carcinoma *in situ*) and although this is not invasive it behaves entirely differently from either Ta or T1 lesions, with approximately 60% of patients developing invasive disease over 5 years if left untreated.

## Treatment of non-muscle-invasive bladder cancer

Bladder TCC is characterised by a tendency to recur in time and space with a variable rate of progression. More than half of all patients will experience recurrence, yet of these recurrences, only 10–15% will develop a subsequent muscle-invasive tumour and therefore acquire a life-threatening disease.

A review in 1996 of combined EORTC and MRC randomised clinical trials in superficial bladder cancer defined low-, intermediate- and high-risk tumours and gave rise to consensus guidelines for the surveillance and management of NMIBC (Pawinski *et al*, 1996). In a more recent combined analysis of 2596 patients from seven EORTC trials, six clinical and pathological factors, number of tumours, tumour size, prior recurrence rate, T category, presence of carcinoma *in situ* and tumour grade, were weighted to derive an algorithm for the calculation of probabilities of recurrence and progression of disease (available at http://www.eortc.be/tools/bladdercalculator) (Sylvester *et al*, 2005).

Further development and validation of this algorithm is likely before its widespread use; however, it highlights the need for such a prognostic tool to individualise the outcome for bladder cancer both in discussing prognosis with patients and as a potential research tool for selecting patients for clinical trials. Furthermore, it may be possible to evaluate and incorporate biomarkers into future algorithms although to date no markers have shown robust clinical utility in this setting.

Primary management of NMIBC involves endoscopic resection of disease followed by intravesical adjuvant therapy. Perioperative intravesical chemotherapy (mitomycin or epirubicin) is effective in reducing tumour recurrence and is considered the standard of care following tumour resection of NMIBC in Europe (NICE, 2002) Prolonged administration of chemotherapy over 6 weeks achieves a further benefit and is recommended for intermediate risk disease. Unfortunately, chemotherapy alone has not been shown to reduce progression and is considered insufficient for NMIBC, which has high risk for recurrence and progression to muscle-invasive disease.

Bacillus of Calmet and Guerin (BCG) is the standard therapy for high-risk NMIBC; BCG delivered intravesically activates a dendritic cell T-helper cell inflammatory response resulting in the infiltration of CD4, CD8 and natural killer cells. For optimal response, BCG is given as a maintenance therapy out to 36 months from diagnosis. A meta-analysis carried out in 2002 confirmed that patients undergoing a maintenance regime had a reduction in the risk of progression, although this effect was modest declining from 14% to a 10% risk (Sylvester *et al*, 2002). A later Cochrane Collaboration review in 2005 comparing BCG with mitomycin-C (an intravesical chemotherapeutic agent) confirmed that BCG reduced recurrence in high-risk cancers but did not demonstrate a definite benefit for progression or survival compared with mitomycin-C (which has not been shown to reduce progression).

## Treatment of muscle-invasive bladder cancer

Approximately 25% of new cases of bladder cancer are muscle-invasive at presentation and up to 20% of initial NMIBC will progress to muscle-invasive. At least 50% of these patients will ultimately progress to metastatic disease. The standard treatment remains radical cystectomy with overall 5-year survival up to 68 and 78% in patients with muscle^-^invasive (pT2 and pT3a) and lymph node-negative disease. Lymph node-positive disease is associated with a significantly worse survival, present in approximately 25% of cases; the overall survival for patients with lymph node-positive disease at 5 years is 25–30% (Stein *et al*, 2001). Cystectomy is a complex procedure requiring urinary diversion and is associated with perioperative complications in up to 30% of patients and a mortality of 2.5% rising to 6% in the United Kingdom. In the period under study, complex pelvic GU surgery was undertaken in district hospitals and more recently there has been a move towards specialist centres served by multidisciplinary teams to manage MIBC. It is too early to determine whether this shift in health-care delivery will have impacted on survival; however, evidence that high-volume specialist centres achieve improved outcomes would suggest that future survival rates will improve. Radiotherapy is considered an alternative to cystectomy and combined radiotherapy plus chemotherapy shows promising results in MIBC, with 5-year survival of 74% in selected series (Shipley *et al*, 2003). In the United Kingdom, an analysis of crude data collected by the BAUS Cancer Registry in 1999 showed no difference in apparent survival or progression in the outcomes of T2 and early T3 disease treated by surgery or radiotherapy (Whelan *et al*, 2002). Only in more advanced T3 disease did surgery appear to have a greater survival benefit but again this was on raw data and the problems of understaging and occult pelvic node disease were not accounted for in this comparison. Few randomised trials have been set up to determine the effect of radiotherapy *vs* cystectomy in the management of MIBC. The lack of quality evidence is the rationale for the Cancer Research UK phase III trial of selective bladder preservation *vs* radical cystectomy (SPARE) in patients with MIBC (UKCRN, 2008).

In the middle of the period under review, effective chemotherapy (methotrexate, vinblastine, adriamycin and cisplatin) against invasive TCC was demonstrated for the first time (Sternberg *et al*, 1989). Subsequent global collaborative trials under the aegis of the EORTC/MRC studied CMV, cisplatin, methotrexate and vinblastine and established a 5% benefit for neoadjuvant chemotherapy (Sternberg and Parmar, 2001). A more recent meta-analysis confirmed the value of neoadjuvant chemotherapy as the standard for muscle-invasive disease (ABC, 2005); however, its utility would have been far too low to impact on survival in the years before 2001.

## Discussion

In the period under study, most treatments for NMIBC and MIBC were delivered by district hospitals. Specialist centres were used exclusively for the delivery of radiotherapy but the continuous follow-up of these patients devolved back to district hospitals. The development of cancer networks with centralised multidisciplinary teams providing high-volume specialist care may change the outcome for patients with MIBC. Within such centres, new standards such as neoadjuvant chemotherapy, surgical teams with high-volume expertise and intensive input to the operative and perioperative management of radical cystectomy (Arumainayagam *et al*, 2008) are available only now and may impact survival in the future.

During the second half of the period under study with a doubling of the number of urological surgeons in England and Wales, more streamlined methods for diagnosing patients with haematuria – ‘The One Stop Haematuria Clinic’ – have been developed for the detection of bladder cancer. The potential to screen at-risk populations has not been realised in part because of the high prevalence of invisible haematuria. It is possible that a genomic risk stratification with incorporation of biomarkers will be effective for the early detection of bladder cancer and there is a need for research in this area in the future.

By far, the largest proportion of incident cases are NMIBC of which high-risk disease will impact most on survival rates. A consistent approach to registration of cases across the United Kingdom will be important as misclassification of carcinoma *in situ* and high-risk Ta has skewed survival data in the past, making clinical correlation and international comparisons difficult. However, within the spectrum of NMIBC, the widespread acceptance of maintenance BCG for high-risk disease has engendered an awareness of aggressive entities within what was termed superficial disease. Although there remains some doubt about the absolute benefit of maintenance therapy, trials of novel agents and delivery systems may begin to impact the outcome for NMIBC. Finally, the consistently worst outcome of women as reported in the survival data is not reflected in prospective clinical trials. Deprivation factors may relate most closely to smoking history and its potential effect on the evolution of the cancer and its prognosis, as well as on increased levels of co-morbidity. But more research is required to understand the causes of the consistently worse outcome of women for survival.

## Conclusion

The lack of change of survival in bladder cancer between the late 1980s and 90s reflects the fact that standard approaches to treatment for invasive bladder cancer remained largely unchanged during that period. The use of chemotherapy in the neoadjuvant, adjuvant and metastatic setting was both too low and too patchy to make any major impact on survival. It is hoped that more consistent and reproducible pathological reporting, more timely use of surgery and radiotherapy and the greater application of neoadjuvant and adjuvant chemotherapy will, in this current decade, have begun to make an impact.
